# Potential Role of PDGFRβ-Associated THBS4 in Colorectal Cancer Development

**DOI:** 10.3390/cancers12092533

**Published:** 2020-09-06

**Authors:** Min Seob Kim, Hyun Seok Choi, Moxin Wu, JiYeon Myung, Eui Joong Kim, Yong Sung Kim, Seungil Ro, Se Eun Ha, Allison Bartlett, Lai Wei, Han-Seung Ryu, Suck Chei Choi, Won Cheol Park, Keun Young Kim, Moon Young Lee

**Affiliations:** 1 Department of Physiology, Digestive Disease Research Institute, and Institute of Wonkwang Medical Science, School of Medicine, Wonkwang University, Iksan 54538, Korea; kmsnim00@naver.com (M.S.K.); wlsahrch006@naver.com (H.S.C.); wumoxinsbsb@163.com (M.W.); mjy1229@naver.com (J.M.); 2 Department of Gastroenterology, Digestive Disease Research Institute, School of Medicine, Wonkwang University, Iksan 54538, Korea; blueliebe98@naver.com (E.J.K.); wms89@hanmail.net (Y.S.K.); hanseung43@naver.com (H.-S.R.); medcsc@wku.ac.kr (S.C.C.); 3 Department of Physiology and Cell Biology, University of Nevada School of Medicine, Reno, NV 89557, USA; sro@medicine.nevada.edu (S.R.); seeunh@med.unr.edu (S.E.H.); allisonbartlett@nevada.unr.edu (A.B.); laiw@med.unr.edu (L.W.); 4 Department of Surgery, Digestive Disease Research Institute, School of Medicine, Wonkwang University, Iksan 54538, Korea; parkwc@wonkwang.ac.kr (W.C.P.); saint9898@naver.com (K.Y.K.)

**Keywords:** THBS4, PDGFRβ, Ca^2+^, colorectal cancer

## Abstract

**Simple Summary:**

We found increased levels of THBS4 and PDGFRb in tumor tissues compared to normal tissues of colon cancer patients. The relationship and the cause of the increase in these proteins had to be determined. Therefore, we performed several experiments and confirmed that excessive PDGFRb stimulation induces the THBS4 secretion through the intracellular Ca^2+^ signaling proteins. Our data show the possibility of post-translational modification of THBS4 by PDGFRb stimulation as there was no significant change in the THBS4 mRNA.

**Abstract:**

Colorectal cancer is a significant cause of death since it frequently metastasizes to several organs such as the lung or liver. Tumor development is affected by various factors, including a tumor microenvironment, which may be an essential factor that leads to tumor growth, proliferation, invasion, and metastasis. In the tumor microenvironment, abnormal changes in various growth factors, enzymes, and cytokines can wield a strong influence on cancer. Thrombospondin-4 (THBS4), which is an extracellular matrix protein, also plays essential roles in the tumor microenvironment and mediates angiogenesis by transforming growth factor-β (TGFβ) signaling. Platelet-derived growth factor receptor β (PDGFRβ), which is a receptor tyrosine kinase and is also a downstream signal of TGFβ, is associated with invasion and metastasis in colorectal cancer. We identified that PDGFRβ and THBS4 are overexpressed in tumor tissues of colorectal cancer patients, and that PDGF-D expression increased after TGFβ treatment in the colon cancer cell line DLD-1. TGFβ and PDGF-D increased cellular THBS4 protein levels and secretion but did not increase THBS4 mRNA levels. This response was further confirmed by the inositol 1,4,5-triphosphate receptor (IP3R) and stromal interaction molecule 1 (STIM1) blockade as well as the PDGFRβ blockade. We propose that the PDGFRβ signal leads to a modification of the incomplete form of THBS4 to its complete form through IP3R, STIM1, and Ca^2+^-signal proteins, which further induces THBS4 secretion. Additionally, we identified that DLD-1 cell-conditioned medium stimulated with PDGF-D promotes adhesion, migration, and proliferation of colon myofibroblast CCD-18co cells, and this effect was intensified in the presence of thrombin. These findings suggest that excessive PDGFRβ signaling due to increased TGFβ and PDGF-D in colorectal tumors leads to over-secretion of THBS4 and proliferative tumor development.

## 1. Introduction

Colorectal cancer (CRC) is one of the most prevalent cancers in the world and is a major cause of cancer-related deaths. During the progression of colorectal adenocarcinomas, gastrointestinal epithelial cells acquire subsequent genetic changes and mutations in specific oncogenic or tumor suppressor genes, which leads to CRC onset, progression, and metastasis [1].

The tumor microenvironment is a tumor pathology-related environment composed of tumor cells, stromal cells, cytokines, immune cells, pericytes, and other components [2,3]. In the tumor microenvironment, several growth factors, proteolytic enzymes, and inflammatory factors act on the surface of tumor cells, which have important effects on cell proliferation, metastasis, and differentiation [4,5]. Thrombospondin-4 (THBS4) is a secreted extracellular matrix protein and one of five members of the thrombospondin protein family. These thrombospondin family members are Ca^2+^-binding extracellular glycoproteins that share a highly conserved C-terminal region but have unique N-terminal domains that play fundamental roles in wound healing and tissue repair [6,7,8,9]. THBS4 is part of a subgroup B of the thrombospondin family members, including THBS3 and 5, and is known to affect intracellular migration, adhesion, and attachment as well as proliferation under varying conditions [10,11,12,13]. There is increasing data to support the role of THBS4 in cancer biology, especially in gastrointestinal and prostate tumors [14,15]. Additionally, it has been suggested that, because of rare genomic alterations in the THBS4 genes, a remarkable activation of THBS4 expression in tumors is most likely regulated through the interaction of invading tumor cells with stromal fibroblasts in the local microenvironment [16]. 

There is a report that platelet-derived growth factor receptor (PDGFR) and THBS4 may be involved in the same pathway [17]. Platelet-derived growth factor receptor (PDGFR) stimulation induces the activation of intracellular signaling pathways that can promote cell migration, invasion, survival, and proliferation [18,19]. PDGFR signaling affects the aggressive behavior of other epithelial tumors such as in breast, liver, and pancreatic cancers, as PDGFR overexpression is associated with advanced-stage cancer and poor prognosis in all tumor types [20,21,22]. In CRC, PDGFR seems to be primarily expressed by stromal cells and pericytes [23,24]. Platelet-derived growth factor receptor β (PDGFRβ), which is a member of the PDGFR family, can also be expressed and induce primary signaling in colorectal tumor cells [25,26]. There are four structurally similar proteins within the PDGF family, namely PDGF-A, PDGF-B, PDGF-C, and PDGF-D, which bind with varying affinities to receptor tyrosine kinase (RTK) receptor units PDGFRα and PDGFRβ [27,28]. Notably, PDGF-D leads to phosphorylation of PDGFRβ, promotes CRC cell migration, invasion, and proliferation, and is highly expressed in human colon cancer DLD-1 cells [29,30].

Several studies have reported that PDGFRβ or THBS4 are associated with transforming growth factor-β (TGFβ) [31,32,33]. Inhibition of TGFβ signaling in tumor cells significantly decreases PDGFRβ expression and PDGF-stimulated tumor cell invasion, which indicates the possibility of downstream TGFβ signaling [33]. In several studies, TGFβ-1, which is an analogous member of the TGFβ cytokine family, promotes angiogenesis by altering the extracellular matrix (ECM) composition through THBS4 upregulation [31,32]. The ability of tumors to manipulate the immune system and allow for uninhibited cell proliferation is partially due to the exploitation of the regulatory cytokine TGFβ signaling pathway. TGFβ is an integral protein involved in cell immune regulation, cell invasion, and microenvironment restructuring [34,35,36]. 

Previous studies show that the phosphorylation of PDGFR activates a signaling cascade hydrolyzing inositol 1,4,5-triphosphate receptor (IP3R), among other proteins, to induce Ca^2+^ release, which leads to a depletion of Ca^2+^ stores within the endoplasmic reticulum (ER) as well as Ca^2+^ re-entering the cell [37,38,39,40]. The IP3R and the stromal interaction molecule 1 (STIM1) proteins are localized to intracellular membranes, such as the ER, and control the influx and diffusion of Ca^2+^ into the cell and cytoplasm. Ca^2+^ is imperative for cellular functions such as intracellular signaling, and IP3R and STIM1 are integral components of the regulatory mechanisms in Ca^2+^ signaling within the cell. Remodeling of Ca^2+^ homeostasis in CRC contributes to proliferation, invasion, and survival [41,42,43]. Based on the studies described above, we hypothesized that increased PDGFRβ induces remodeling of Ca^2+^ homeostasis, and, thereby, increases THBS4 secretion.

In this study, using cell assay techniques, transcript and protein analyses in colon cancer patient tissue, and the cell line, we show that PDGFRβ stimulation by TGFβ and PDGF-D increases the THBS4 secretion via IP3R and STIM1 in colorectal cancer.

## 2. Results

### 2.1. Expression of THBS4 and PDGFRβ in Colorectal Cancer

**Figure 1:**
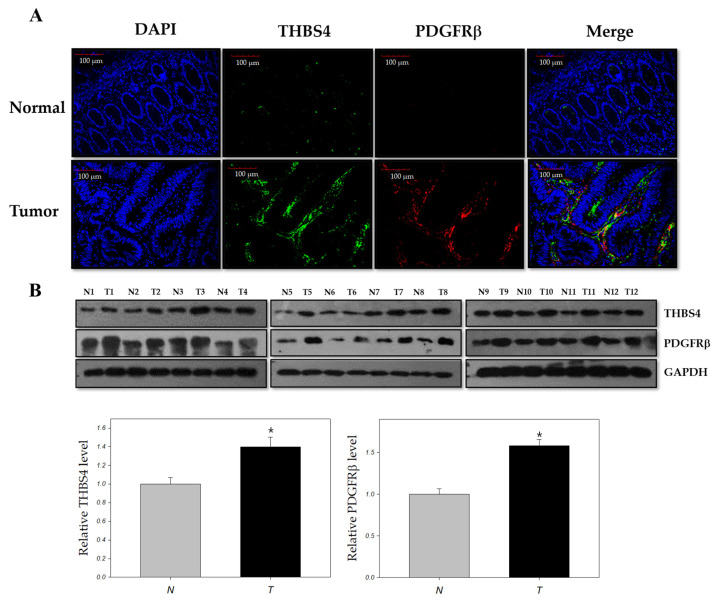
Co-immunofluorescence and immunoblots for Thrombospondin-4 (THBS4) and Platelet-derived growth factor receptor β (PDGFRβ) in normal and tumor tissues of colon cancer patients. (**A**) Immunofluorescence with an anti-THBS4 antibody (green) and anti-PDGFRβ antibody (red) on normal (N) and tumor (T) tissues of colon cancer patients. Scale bars = 100 µm. (**B**) Western blot with anti-THBS4 and anti-PDGFRβ antibodies on normal and tumor tissues of colon cancer patients. * *p* < 0.05, compared with normal tissue, *t-*test.

There is increasing evidence that THBS4 and PDGFRβ are associated with tumor development [10,11,12,13,16,20,21,22,23,24,25,26]. To investigate the association between these two proteins in the tumor, we performed co-immunofluorescence and Western blot analyses for THBS4 and PDGFRβ in tumor tissues of patients with CRC. Compared to normal tissues, elevated levels of PDGFRβ and THBS4 were found in tumor tissues, which confirms our hypothesis that these two proteins are significantly associated with tumor development in CRC (Figure 1 and Figure S1). 

### 2.2. TGFβ Stimulates Increased mRNA and Protein Expression of PDGF-D

**Figure 2:**
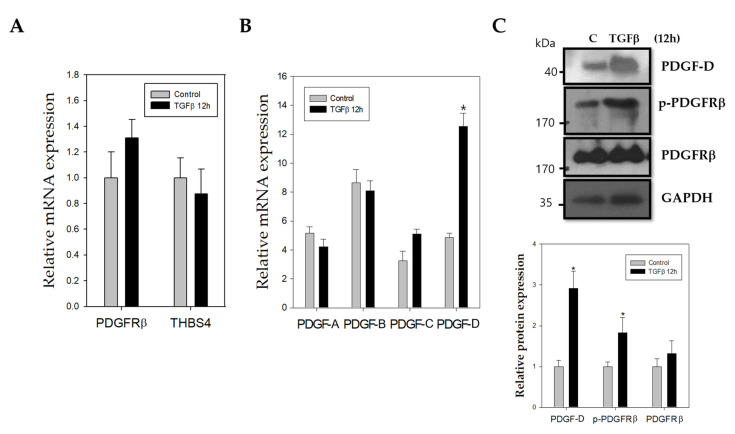
Effect of TGFβ on mRNA levels of THBS4, PDGFRβ, and PDGFRβ ligands. Relative mRNA expression levels as determined with real-time polymerase chain reaction (PCR) for (**A**) PDGFRβ, THBS4, (**B**) PDGF-A, PDGF-B, PDGF-C, and PDGF-D of DLD-1 cells cultured in the presence (TGFβ 10mM) or absence (control) of TGFβ for 12 h. (**C**) Western blot with anti-PDGF-D, p-PDGFRβ, and PDGFRβ antibodies in DLD-1 cells cultured in the presence (TGFβ 10 μM) or absence (control) of TGFβ for 12 h. Three independent experiments were performed in duplicate. * *p* < 0.05 when compared with the control *t-*test.

The role of TGFβ in CRC is also of interest, as it has been shown to be an upstream signal of PDGFRβ in tumor cells [33], and is involved in angiogenesis through THBS4 [31,32]. Therefore, to investigate the effects of TGFβ on PDGFRβ and THBS4 overexpression in CRC, we examined the mRNA levels of PDGFRβ and THBS4 after treatment with TGFβ for 12 h in DLD-1 cells, which is a colon cancer cell line. Neither THBS4 nor PDGFRβ mRNA levels were significantly altered in response to TGFβ treatment (Figure 2A). However, PDGF-D mRNA levels, among PDGFR ligands (PDGF-A, PDGF-B, PDGF-C, and PDGF-D), were significantly increased by TGFβ (Figure 2B). The protein expression level of PDGF-D also increased, and a PDGF-D receptor, [44] PDGFRβ, was activated in the presence of TGFβ (Figure 2C). These results were similar in another CRC cell line HCT-116 (Figure S2).

### 2.3. TGFβ and PDGF-D Influence the Post-Translational Modification and Secretion of THBS4

**Figure 3:**
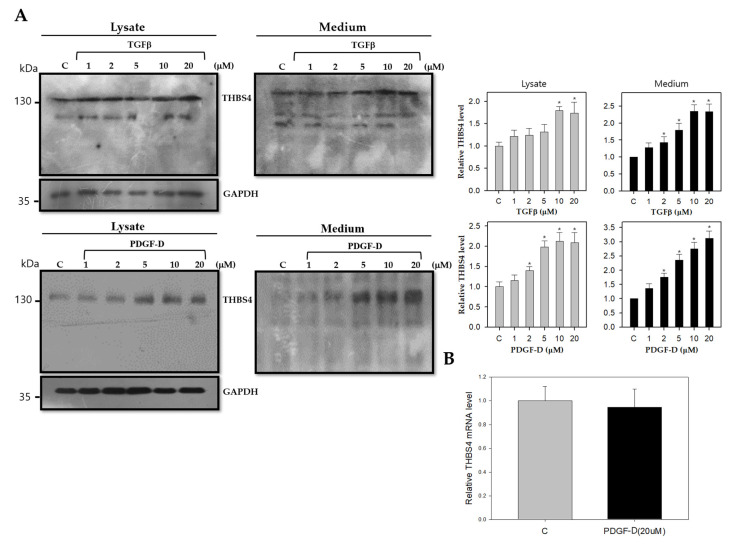
Effect of TGFβ or PDGF-D on THBS4 of lysate and cultured medium of DLD-1 cells. (**A**) Western blot with anti-THBS4 antibody in whole cell lysate (left panel) or cultured medium (right panel) of DLD-1 cells cultured in the presence of TGFβ (1, 2, 5, 10 and 20 μM) for 20 h or PDGF-D (1, 2, 5, 10, and 20 μM) for 8 h. (**B**) Relative mRNA expression levels as determined with real-time polymerase chain reaction (PCR) for THBS4 of DLD-1 cells cultured in the presence (black) or absence (gray) of PDGF-D (20 μM) for 8 h. Three independent experiments were performed in duplicate. * *p* < 0.05 when compared with the control *t-*test.

Based on the above results, we hypothesized that PDGF-D would act as a downstream signal for TGFβ on THBS4 upregulation. To examine how TGFβ and its downstream protein PDGF-D affects THBS4 protein levels and secretion, since TGFβ did not significantly alter THBS4 mRNA, DLD-1 cells were treated with TGFβ at 1, 2, 5, 10, and 20 μM for 20 h or PDGF-D for 8 h. Cell lysates and media containing THBS4 were considered to be intracellular and extracellular, respectively. Increased levels of THBS4 were found in the cell lysate and culture medium in the presence of TGFβ or PDGF-D in a dose-dependent manner (Figure 3A). However, PDGF-D did not significantly alter THBS4 mRNA levels like TGFβ (Figure 3B). Thus, these data suggest that TGFβ and PDGF-D in DLD-1 cells might be involved in the post-translational modification and secretion of THBS4, rather than conferring protein production through THBS4 mRNA expression.

### 2.4. Knockdown of Ca^2+^ Signaling Proteins Decreases THBS4 Secretion even on PDGF-D

**Figure 4:**
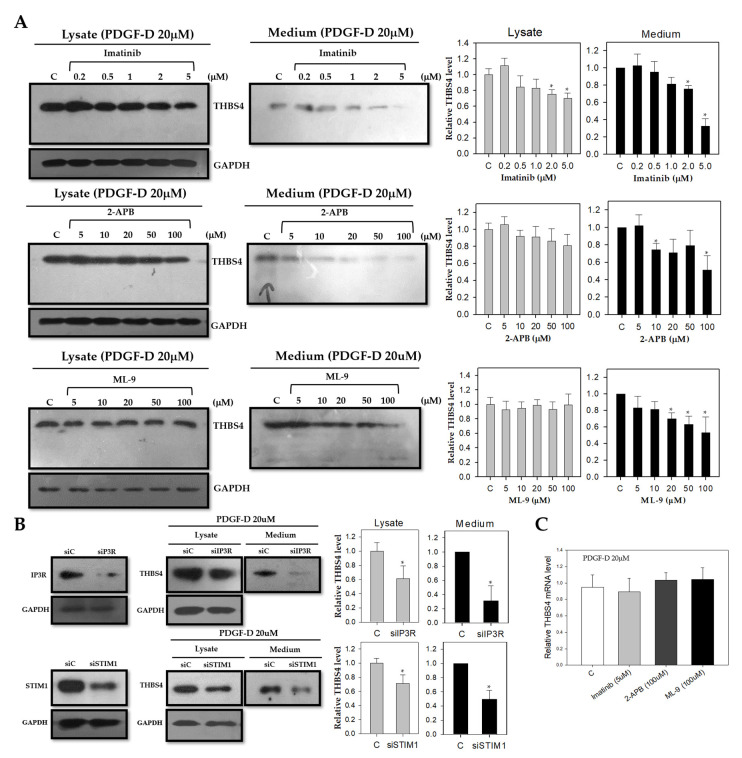
Effect of PDGF-D stimulation of THBS4 after blockage of PDGFRβ, IP3R, and STIM1. (**A**) Western blot with anti-THBS4 antibody in whole cell lysate (left panel) or cultured medium (right panel) of DLD-1 cells cultured with PDGF-D (20 μM) for 8 h in the presence of imatinib (0, 0.2, 0.5, 1, 2, and 5 μM) for 16 h, 2-APB (0, 5, 10, 20, 50, and 100 μM) for 16 h, or ML-9 (0, 5, 10, 20, 50, and 100 μM) for 16 h, respectively. (**B**) Western blot with anti-THBS4 antibody in whole cell lysate or cultured medium of DLD-1 cells transfected with siIP3R or siSTIM1 and cultured with PDGF-D (20 μM) for 8 h. (**C**) Relative mRNA expression levels as determined with real-time PCR for THBS4 of DLD-1 cells cultured with PDGF-D (20 μM) for 8 h in the presence of imatinib (5 μM) for 16 h, 2-APB (100 μM) for 16 h or ML-9 (100 μM) for 16 h, respectively. Three independent experiments were performed in duplicate. * *p* < 0.05 when compared with the control *t-*test.

To investigate the effects of PDGF-D on the secretion of THBS4, we treated DLD-1 cells with the PDGFRβ inhibitor, imatinib, to identify changes in THBS4 secretion. By inhibiting PDGFRβ, PDGF-D signaling was also subsequently inhibited. DLD-1 cells were treated with imatinib at 0.2, 0.5, 1, 2, and 5 μM, respectively, for 16 h, including an additional treatment with PDGF-D for 8 h. In both the cell lysate and culture medium, there was decreased expression of THBS4 in a dose-dependent manner, which indicates the essential role of PDGF-D signaling on THBS4 expression (Figure 4A, upper panel).

Previous literature shows a Ca^2+^ binding domain in the C-terminal region of THBS4 [6,7,8,9]. Therefore, we also suspected that the Ca^2+^ signaling proteins, IP3R and STIM1, might be involved in THBS4 activity. To investigate this idea, we treated DLD-1 cells with the IP3R inhibitor, 2-APB, and a STIM1 inhibitor, ML-9, at concentrations of 5, 10, 20, 50, and 100 μM for 16 h. We also administered an additional treatment with PDGF-D for 8 h. Similar to PDGFRβ inhibition, we found decreased expression of THBS4 in the culture medium under 2-APB or ML-9 (Figure 4A, middle and lower panel). Despite rescue treatment with PDGF-D in siIP3R-transfected and siSTIM1-transfected DLD-1 cells, THBS4 levels remained at reduced levels in the lysate and cultured medium (Figure 4B). However, even with the treatment of inhibitors such as imatinib, 2-APB, and ML-9, there was no significant decrease in THBS4 mRNA expression (Figure 4C). These data suggest that, not only PDGFRβ, but also IP3R and STIM1 participate in the post-translational modification and secretion of THBS4.

### 2.5. PDGF-D Increases THBS4 through PDGFRβ and Ca^2+^ Signaling Proteins

**Figure 5:**
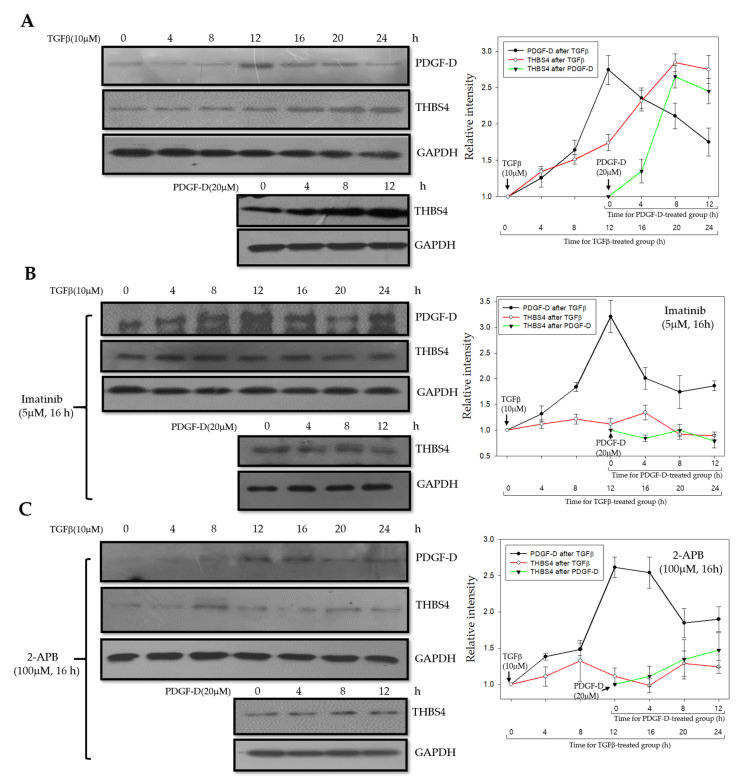
Effect of imatinib and 2-APB on THBS4 regulation by TGFβ and PDGF-D in a time-dependent manner. (**A**) Western blot with anti-PDGF-D and anti-THBS4 antibodies in the presence of TGFβ 10 µM (0, 4, 8, 12, 16, 20, and 24 h) or with anti-THBS4 antibody in the presence of PDGF-D 20 µM (0, 4, 8 and 12 h) in DLD-1 cells. (**B**) Western blot with anti-PDGF-D and anti-THBS4 antibodies in the presence of TGFβ 10 µ M (0, 4, 8, 12, 16, 20, and 24 h) or with anti-THBS4 antibody in the presence of PDGF-D 20 µM (0, 4, 8, and 12 h) in DLD-1 cells cultured with imatinib 5 µM for 16 h. (**C**) Western blot with anti-PDGF-D and anti-THBS4 antibodies in the presence of TGFβ 10 µM (0, 4, 8, 12, 16, 20, and 24 h) or with anti-THBS4 antibody in the presence of PDGF-D 20 µM (0, 4, 8 and 12 h) in DLD-1 cells cultured with 2-APB 100 µM for 16 h. The graphs in the right panel represent the relative expression of PDGF-D (black line with closed circle). THBS4 (red line with open circle) in relation to exposure (time, h) with TGFβ and the relative expression of THBS4 after the addition of PDGF-D (green line with closed triangle) through 12 h. Three independent experiments were performed in duplicate.

Previously, we confirmed that PDGFRβ and Ca^2+^ signaling proteins mediate changes in THBS4 through PDGF-D stimulation. However, to validate that TGFβ increases PDGF-D, and, ultimately, affects THBS4, we treated DLD-1 cells with TGFβ for 0, 4, 8, 12, 16, 20, and 24 h and identified the expression levels of PDGF-D and THBS4. We found that, after TGFβ treatment in DLD-1 cells, the relative intensity of PDGF-D peaked at 12 h and gradually decreased over time, while, that of THBS4, increased from 12 h to 20 h. We treated the same cells with PDGF-D for 12 h and found that the THBS4 relative intensity peaked at 8 h after PDGF-D treatment. This was consistent with the highest expression of PDGF-D at 12 h after TGFβ treatment (Figure 5A). Results of TGFβ treatment in the presence of the PDGFRβ-inhibitor, imatinib, showed an elevated relative intensity level for PDGF-D at 12 h after treatment, which is similar to that of cells without the addition of imatinib. However, THBS4 levels remained low after the addition of PDGF-D in imatinib-treated cells, which indicates the significance of PDGFRβ on THBS4 upregulation (Figure 5B). TGFβ treatment in cells cultured with 2-APB also showed increased PDGF-D expression at 12 h but did not significantly alter THBS4 levels nor did the addition of PDGF-D have any significant effect on THBS4 (Figure 5C). These results provide further evidence that TGFβ increases PDGF-D and PDGF-D subsequently increases THBS4 through PDGFRβ in a sequential manner, and Ca^2+^ signaling proteins also function as in our proposed mechanism. 

### 2.6. Stimulation of PDGFRβ in Colon Cancer Cells Promotes Adhesion, Migration, and Proliferation of Colonic Myofibroblasts

**Figure 6:**
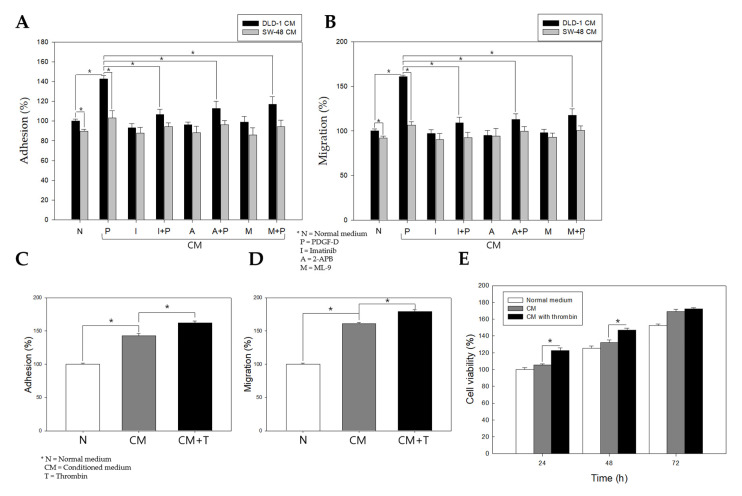
Effect of DLD-1 cell conditioned medium (CM) stimulated with PDGF-D and additional thrombin treatment on CCD-18co cells. (**A**,**B**) Adhesion and migration of CCD-18co cells cultured with DLD-1 CM stimulated with PDGF-D (P), imatinib (I), I + P, 2-APB (A), A + P, ML-9 (M), and M + P. DLD-1 cells were treated with imatinib 5 M, 2-APB 100 or ML-9 100 for 16 h, and then PDGF-D 20 additionally treated for 8 h. (**C**,**D**,**E**) CCD-18co cells were cultured with CM or CM with thrombin (1 U/mL) (CM + T) of DLD-1 cells stimulated with PDGF-D 20 for 8 h and subjected to an adhesion assay, migration assay, and an 3-(4,5-dimethylthiazol-2-yl)-2,5-diphenyl tetrazolium bromide (MTT) assay. All experiments were performed in duplicate in three independent experiments. * *p* < 0.05, compared with normal or CM *t-*test.

To investigate how THBS4 is stimulated and secreted by colon cancer cells, and whether the colon cancer cells can affect neighboring cells around it, colonic myofibroblast CCD-18co cells were cultured in the conditioned medium (CM) of DLD-1 cells or SW-48 cells stimulated with respective factors (Figure 6A,B). SW-48 cells are colorectal adenocarcinoma cells like DLD-1, but do not express THBS4 (Figure S4) [45]. Adhesion and migration assays were also performed on the CCD-18co cells. The PDGF-D-stimulated DLD-1 CM increased adhesion and migration compared to that in normal medium. However, PDGF-D-stimulated DLD-1 CM treated with imatinib while 2-APB or ML-9 did not. Conversely, the PDGF-D-stimulated SW-48 CM did not significantly increase adhesion or migration of CCD-18co cells (Figure 6A,B). This implies that THBS4 secreted by PDGF-D stimulation in DLD-1 cells might promote adhesion and migration of CCD-18co cells. Additionally, these properties were further enhanced with the inclusion of thrombin in the CM (Figure 6C–E). These results suggest that excessive stimulation of PDGFRβ in colon cancer cells may over-secrete THBS4 and promote the adhesion, migration, and proliferation of normal colon myofibroblasts.

### 2.7. A Proposed Novel Molecular Pathway for the Development of Colon Cancer

**Figure 7:**
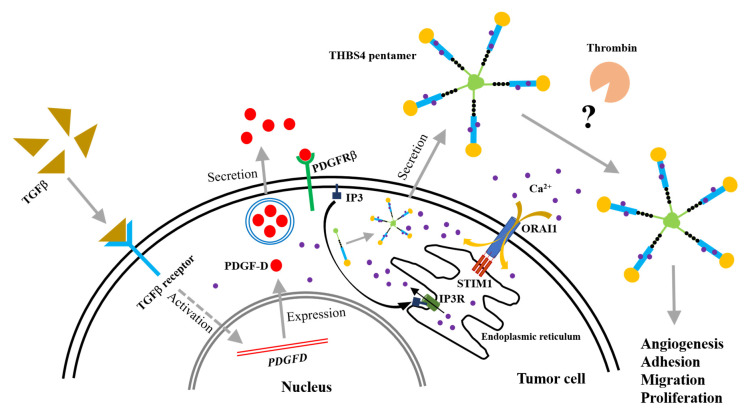
Model representing possible molecular pathways by which PDGFRβ and THBS4 cause the development of colorectal cancer.

Based on these results, we hypothesize: (1) TGFβ secreted by CRC cells activates TGFβ receptors in tumor cells and increases expression of PDGF-D, (2) secreted PDGF-D subsequently activates PDGFRβ to be overexpressed in tumor cells through autocrine or paracrine mechanisms. Following this, PDGFRβ activates the IP3 signaling molecule in the plasma membrane to stimulate its corresponding Ca^2+^ release channel, IP3R, from the ER. (3) Following the functioning of IP3R, the Ca^2+^ inside the ER is released into the cytoplasm, and through Ca^2+^ binding, the incomplete form of THBS4 is transformed into the complete THBS4 pentamer in the ECM. (4) THBS4 is secreted into the extracellular space and induces angiogenesis, adhesion, migration, and proliferation of tumor tissue, which is further promoted by thrombin. (5) Lastly, this response leads to a partial depletion of Ca^2+^ inside the ER, which further increases the secretion of THBS4 through STIM1, as well as intracellular Ca^2+^ influx into the cytoplasm (Figure 7).

## 3. Discussion

In this study, we examined the relationship among PDGFRβ, THBS4, and CRC. This study showed that PDGFRβ was overexpressed and that THBS4 was significantly increased in the tumor tissue when compared to normal colon tissue in CRC patients (Figure 1). We believe that PDGFRβ and THBS4 are involved in the progression of tumors that have already formed rather than tumorigenesis.

The role of THBS4 in cancer has primarily been focused on tumor adhesion, migration, invasion, and angiogenesis [14,15,16,31,32] while the role of PDGFRβ has been extensively studied in epithelial-mesenchymal transition (EMT) and metastasis [25,26,33,46,47]. These two proteins have been shown to be associated with TGFβ in cancer. However, specific mechanisms remain undiscovered, and several studies indicate that TGFβ inhibits early-stage tumors by inducing cancer cell apoptosis and cell cycle arrest and by promoting cell differentiation. However, TGFβ has been found to promote tumor growth and metastasis at a later stage by activating specific pathways in vascular cell types and by increasing angiogenesis [32,48]. Due to compelling evidence presented in previously published work enforcing our original hypothesis, we sought to identify the roles of PDGFRβ and THBS4 using TGFβ. TGFβ treatment increased PDGF-D in DLD-1 cells while TGFβ and PDGF-D increased protein levels and secretion of THBS4 (Figure 2 and Figure 3). PDGF-D functions by binding to PDGFRβ [27,49] and promoting cell growth, which increases the aggressiveness of other cancer cells, angiogenesis, and EMT of CRC [29]. In our study, TGFβ increased mRNA levels of PDGF-D (Figure 2B and Figure S2B), but PDGF-D did not increase the mRNA levels of THBS4 (Figure 3B). This implies that PDGFRβ activation by PDGF-D occurs upstream of THBS4 secretion and that TGFβ is located upstream of PDGFRβ activation, which suggests that both TGFβ and PDGFRβ are involved in stabilization through post-translational modification of THBS4. However, these responses were reduced by blocking PDGFRβ, IP3R, and STIM1 (Figure 4A,B), which suggests that IP3R and STIM1 may also be involved in the stabilization of THBS4. Considering the possibility that the increase in THBS4 was not increased by PDGF-D, but by each specific inhibitor and siRNAs, we checked the THBS4 levels without PDGF-D stimulation. There was no significant change in THBS4 without PDGF-D stimulation (Figure S3). Additionally, the inhibitors and siRNAs did not significantly alter the proliferation of DLD-1 cells without PDGF-D stimulation, which indicates that the THBS4 decrease was not due to cell death (Figure S5 and S6). Time-dependent experiments supplemented this hypothesis. After TGFβ treatment, PDGF-D increased first, which was followed by THBS4. Based on this, PDGF-D treatment ultimately increased THBS4. Additionally, TGFβ treatment increased PDGF-D but did not increase THBS4 in cells with imatinib and 2-APB in a time-dependent manner (Figure 5). Since imatinib blocks PDGFRβ and PDGF-D expression by TGFβ does not affect the regulation of THBS4, blocking IP3R with 2-APB affects THBS4 secretion after PDGFRβ activation. We describe in this scenario that stability of THBS4 by the PDGFRβ pathway based off of the evidence provided indicated increased protein and secretion levels, despite no significant changes in mRNA. However, other post-translational modifications, such as protein folding, may be involved in this pathway, which should be examined further.

Abnormal Ca^2+^ signaling by altered channel expression or activation contributes to carcinogenesis and promotes tumor development [50]. The Ca^2+^ concentration in the cytoplasm remains very low but can be temporarily increased by IP3R and store-operated Ca^2+^ entry (SOCE) [51,52]. The collapse of normal Ca^2+^ signaling contributes to the development of malignant phenotypes. Tumors reconstitute their Ca^2+^ signaling networks to proliferate at high rates, increase cell motility and invasion, manipulate the immune response, or cause angiogenesis [53,54,55,56,57,58]. Our data demonstrate the possibility that excessive PDGF-D levels may disrupt the homeostasis of Ca^2+^ signaling and promote the excessive secretion of THBS4. This phenomenon might be caused by the Ca^2+^-binding domain of THBS4 [6,7,8,9], and secreted THBS4 is known to regulate the tumor microenvironment through adhesion, migration, and attachment functions of ECM proteins [10,16]. We found that the conditioned medium of PDGF-D-stimulated DLD-1 cells increased adhesion, migration, and proliferation of normal colon myofibroblasts. This became more apparent through SW-48 cells that do not express THBS4 [45] and various CM that used imatinib, 2-APB, and ML-9 together (Figure 6). These results indicate that PDGF-D affects myofibroblasts by altering the microenvironment around the tumor with THBS4, which might be induced to convert to cancer-associated fibroblasts. This response was further increased when the CM contained thrombin (Figure 6C–E), as it may have directly affected myofibroblasts. However, there remains the possibility of thrombin promoting the action of THBS4, and, additionally, one of the members of the THBS family, THBS1, has also been shown to be sensitive to thrombin in platelets secreted and cleaved by thrombin [59,60,61]. While the results presented in this paper are promising, the role of thrombin in THBS4 remains to be established and needs to be explored in future studies.

Abnormal Ca^2+^ signaling by altered channel expression or activation contributes to carcinogenesis and promotes tumor development [50]. The Ca^2+^ concentration in the cytoplasm remains very low but can be temporarily increased by IP3R and store-operated Ca^2+^ entry (SOCE) [51,52]. The collapse of normal Ca^2+^ signaling contributes to the development of malignant phenotypes. Tumors reconstitute their Ca^2+^ signaling networks to proliferate at high rates, increase cell motility and invasion, manipulate the immune response, or cause angiogenesis [53,54,55,56,57,58]. Our data demonstrate the possibility that excessive PDGF-D levels may disrupt the homeostasis of Ca^2+^ signaling and promote the excessive secretion of THBS4. This phenomenon might be caused by the Ca^2+^-binding domain of THBS4 [6,7,8,9], and secreted THBS4 is known to regulate the tumor microenvironment through adhesion, migration, and attachment functions of ECM proteins [10,16]. We found that the conditioned medium of PDGF-D-stimulated DLD-1 cells increased adhesion, migration, and proliferation of normal colon myofibroblasts. This became more apparent through SW-48 cells that do not express THBS4 [45] and various CM that used imatinib, 2-APB, and ML-9 together (Figure 6). These results indicate that PDGF-D affects myofibroblasts by altering the microenvironment around the tumor with THBS4, which might be induced to convert to cancer-associated fibroblasts. This response was further increased when the CM contained thrombin (Figure 6C–E), as it may have directly affected myofibroblasts. However, there remains the possibility of thrombin promoting the action of THBS4, and, additionally, one of the members of the THBS family, THBS1, has also been shown to be sensitive to thrombin in platelets secreted and cleaved by thrombin [59,60,61]. While the results presented in this paper are promising, the role of thrombin in THBS4 remains to be established and needs to be explored in future studies.

## 4. Materials and Methods 

### 4.1. Cell Culture and Treatments

Human colon cancer epithelial cells DLD-1 (Male, Dukes’s stage C), HCT116 (Male, Dukes’s stage D), and colonic myofibroblast CCD-18co cells (Female) were obtained from the Korean Cell Line Bank, and SW48 (Female, Dukes’s stage C) was purchased from ATCC. DLD-1 and HCT116 cells were cultured in RPMI-1640 (Gibco, Carlsbad, CA, USA) and CCD-18co and SW48 were cultured in Dulbecco’s Modified Eagle Medium (DMEM) (Gibco, Carlsbad, CA, USA) with L-glutamine (300 mg/L) supplemented with 10% fetal bovine serum (Gibco), penicillin (100 U/mL), and streptomycin (100 μM). DLD-1 cells were treated with TGFβ (R&D systems, Minneapolis, MN, USA), PDGF-D (R&D systems), imatinib (Tocris Bioscience, Bristol, UK), 2-APB (Tocris), and ML-9 (Sigma Aldrich, St. Louis, MO, USA) for 8 or 16 h. The conditioned medium was obtained from the supernatant of cells collected 8 h after PDGF-D stimulation, and treated with fresh medium in a 1:1 ratio to CCD-18co cells. To prevent mycoplasma contamination, Plasmocin™ (Invitrogen, San Diego, CA, USA, ant-mpp) was added to the medium.

### 4.2. Human Tissue

Surgically resected normal and tumor tissues from 12 sigmoid or rectal cancer patients were obtained from Wonkwang University Hospital (Table 1). Informed consent was obtained from all patients. The research approval number is WKIRB-201911-BR-086. 

### 4.3. Transfection 

DLD-1 cells were transfected with IP3R siRNA 10nM (sc-42475, Santa Cruz Biotechnology, Santa Cruz, Dallas, TX, USA) or STIM1 siRNA 10 nM (sc-76589) along with control siRNA (sc-37007). IP3R and STIM1 siRNA are pools of three target-specific 19–25 nt siRNAs. The transfection reagent (sc-29528) was used per the manufacturer’s protocol. After 24 h of transfection, the medium was replaced by fresh medium. After 24 h, the cells were incubated with PDGF-D for 8 h.

### 4.4. Western Blot Analysis

Cells were lysed in a protein extraction solution (Intron, Seongnam, South Korea). After centrifugation, the samples were boiled at 95 °C for 10 min, and 50 µg protein of each lysate was subjected to electrophoresis on 10% sodium dodecyl sulfate polyacrylamide gels. All samples were electroblotted on polyvinylidene fluoride membranes (Merck Millipore, Burlington, MA, USA) and, after blocking, the blots were incubated with appropriate rabbit anti-THBS4 (Santa Cruz Biotechnology, sc-390734), rabbit anti-PDGFRβ (Abcam, Cambridge, MA, USA, ab32570), mouse anti-phosphorylated PDGFRβ (Santa Cruz Biotechnology, sc-365464), rabbit anti-PDGF-D (Abcam, Cambridge, MA, USA, ab234666), mouse anti-IP3R (Santa Cruz Biotechnology, sc-271197), and rabbit anti-STIM1 (Cell Signaling Technology, Danvers, MA, USA, 5668S) antibodies in TBS-T (TBS-0.05% Tween 20) for 90 min, which was followed by washing three times with TBS-T for 15 min each, and incubation with horseradish peroxidase-conjugated anti-mouse or rabbit immunoglobulin G antibodies for 1 h. After further washing, the blots were incubated for 3 min with Western blotting HRP-substrate (Merck Millipore), and chemiluminescence was detected after exposure of the filters to ECL-Western blot films for 10 s to 10 min. Original Western blots are shown on Figures S7–S14.

### 4.5. Trichloroacetic Acid Precipitation

Supernatants were precipitated with 10% trichloroacetic acid (TCA) and incubated on ice for 1 h before centrifugation at 18,000 × *g* for 30 min. The protein pellets were washed three times with cold acetone and centrifuged at 18,000× *g* for 5 min. TCA-precipitated proteins were separated using Western blot analysis.

### 4.6. Immunofluorescence

After surgical resection of tissues, the tissues were fixed immediately with 4% paraformaldehyde for 4 h. After washing three times with phosphate-buffered saline (PBS), the tissues were fixed with acetone for 15 min. After washing three times with PBS, the tissues were dehydrated in 30% sucrose until it subsided. The tissues were then frozen with Frozen Section Compound (Leica Biosystems Richmond Inc, Richmond, IL, USA). The cryostat-sectioned human colon tissues were blocked at room temperature for 1 h in diluted egg white with TBS (1 egg white: 100 mL TBS) to block endogenous biotin and 1 h in 4% skimmed milk containing 0.1% Triton X-100. Primary antibodies against the following antigens were applied overnight: anti-THBS4 (mouse, 1:50, Santa Cruz Biotechnology, Santa Cruz, CA, USA, sc-390734) and anti-PDGFR (rabbit, 1:100, Abcam, Cambridge, MA, USA, ab32570). The tissues were incubated with biotin for 1 h at room temperature and then with Alexa488-conjugated antibodies or Alexa594-conjugated streptavidins for 2 h at room temperature. Images were collected using confocal microscopy and the Fluoview FV10-ASW 3.1 Viewer software (Olympus, Tokyo, Japan) with an Olympus FV1000 confocal laser scanning microscope (Olympus).

### 4.7. Migration Assay

CCD-18co cells were trypsinized, resuspended in serum-free DMEM, and plated into the upper chambers of the Boyden chamber assay with PDGF-D-stimulated DLD-1 cell-conditioned medium. The lower chambers were plated with 10% fetal bovine serum (FBS). Cells were then incubated at 37 °C for 4 h and the lower surface of polycarbonate membranes was stained with 0.1% crystal violet. Lastly, the number of cells was quantified.

### 4.8. Proliferation Assay

The proliferation assay was performed using 3-(4, 5-dimethylthiazol-2-yl)-2,5-diphenyltetrazolium bromide (MTT) (Sigma Aldrich, St. Louis, MO, USA). CCD-18co cells were cultured for 24, 48, and 72 h with 10% FBS, washed with PBS, and incubated for 4 h at 37 °C with 0.5 mg/mL MTT solution. After incubation, the absorbance was measured with a microplate reader (ReTiSoft Inc., Mississauga, ON, Canada) at a wavelength of 570 nm to determine cell proliferation.

### 4.9. Adhesion Assay

The adhesion assay was performed using collagen I-coated plates. The cells were suspended in serum-free medium, transferred to coated wells, and incubated at 37 °C in 5% CO_2_ for 2 h. After incubation, the plates were washed with PBS to remove unbound cells. The cells were then stained with 0.1% crystal violet, and the absorbance was measured with a microplate reader (ReTiSoft Inc.) at a wavelength of 570 nm to determine the number of cells attached to the surface.

### 4.10. Quantitative Reverse Transcription-PCR (qRT-PCR)

RNA was isolated from cells with Trizol (Invitrogen, Carlsbad, CA, USA), according to the manufacturer’s instructions and amplified by PCR using the POWER SYBR^®^ Green PCR Master Mix (Thermo Fisher Scientific, Waltham, MA, USA), according to the manufacturer’s instructions. Each sample was tested in triplicate. Glyceraldehyde 3-phosphate dehydrogenase (GAPDH) was used as an endogenous control. Primer sequences are shown in Table 2.

### 4.11. Statistical Analysis

All blots were analyzed using ImageJ software 1.53. All statistical analyses were performed using Student’s t-tests with * *p* < 0.5 considered as significant using 10.0 SigmaPlot software (Systat Software, Inc., San Jose, CA, USA). The data are expressed as mean ± standard error (SE).

## 5. Conclusions

Our data suggest that the secretion pathway of THBS4 by activating PDGFRβ following TGFβ in CRC involves Ca^2+^ signaling proteins such as IP3R and STIM1 in this pathway (Figure 7). Since THBS4 may increase tumor invasion by regulating the microenvironment through this pathway, which determines the function of THBS4 may significantly contribute to understanding the development of CRC in future research and lead to potential pharmacological agents in hopes of attenuating cancer development.

## Figures and Tables

**Table 1:** Information on 12 colorectal cancer (CRC) patients.

Sex	Age	Location
Male	79	Rectum
Male	57	Rectum
Male	88	Sigmoid
Male	65	Sigmoid
Male	35	Rectum
Male	82	Rectosigmoid
Male	46	Descending colon
Female	77	Sigmoid
Female	41	Sigmoid
Female	76	Rectum
Female	59	Rectosigmoid
Female	79	Rectum

**Table 2:** The sequence of primers.

Gene	Sequence (5’ → 3’)
PDGF-A	Forward: CGTAGGGAGTGAGGATTCTTTG Reverse: AAATGACCGTCCTGGTCTTG
PDGF-B	Forward: CTCGATCCGCTCCTTTGATG Reverse: AGGAAGTTGGCGTTGGTG
PDGF-C	Forward: GTCAATGTGTCCCAAGCAAAG Reverse: CCACGTCGGTGAGTGATTT
PDGF-D	Forward: GAAATTGTGGCTGTGGAACTG Reverse: GGCCAGGCTCAAACTGTAATA
PDGFR	Forward: GTGACAGACTACCTCTTTGG Reverse: CTACATCTCCCAGTGTCTCC
THBS4	Forward: GTTCAGCCACCATCTTCGGTC Reverse: GCACCTTCCCATCGTTCTTCAG
GAPDH	Forward: CCACATCGCTCAGACACCATG Reverse: GTCAATGAAGGGGTCATTGATGGC

## Supplementary Materials

The following are available online at https://www.mdpi.com/2072-6694/12/9/2533/s1. Figure S1. Co-immunofluorescence and immunoblots for THBS4 and PDGFRβ in normal and tumor tissues of colon cancer patients, Figure S2. Effect of TGFβ on mRNA levels of THBS4, PDGFRβ, and PDGFRβ ligands, Figure S3. Protein levels of THBS4 without PDGF-D stimulation, Figure S4. Basal THBS4 levels in DLD-1, SW40, and HCT-116 cells, Figure S5. Cell proliferation of DLD-1 cells after treatment with imatinib, 2-APB, and ML-9, Figure S6. Cell proliferation of DLD-1 cells after siIP3R and siSTIM1 transfection, Figure S7. Original Western Blots of Figure 1, Figure S8. Original Western Blots of Figure 2, Figure S9. Original Western Blots of Figure 3, Figure S10. Original Western Blots of Figure 4A, Figure S11. Original Western Blots of Figure 4B, Figure S12. Original Western Blots of Figure 5A, Figure S13. Original Western Blots of Figure 5B, Figure S14. Original Western Blots of Figure 5C.

## References

[1] De Rosa M., Pace U., Rega D., Costabile V., Duraturo F., Izzo P., Delrio P. (2015). Genetics, diagnosis and management of colorectal cancer (Review). Oncol. Rep..

[2] Castells M., Thibault B., Mery E., Golzio M., Pasquet M., Hennebelle I., Bourin P., Mirshahi M., Delord J.P., Querleu D. (2012). Ovarian ascites-derived Hospicells promote angiogenesis via activation of macrophages. Cancer Lett..

[3] Guo S., Deng C.-X. (2018). Effect of stromal cells in tumor microenvironment on metastasis initiation. Int. J. Biol. Sci..

[4] Castells M., Thibault B., Delord J.P., Couderc B. (2012). Implication of tumor microenvironment in chemoresistance: Tumor-associated stromal cells protect tumor cells from cell death. Int. J. Mol. Sci..

[5] Meads M.B., Gatenby R.A., Dalton W.S. (2009). Environment-mediated drug resistance: A major contributor to minimal residual disease. Nat. Rev. Cancer.

[6] Adams J.C. (2001). Thrombospondins: Multifunctional regulators of cell interactions. Annu. Rev. Cell Dev. Biol..

[7] Adams J.C., Lawler J. (2004). The thrombospondins. Int. J. Biochem. Cell Biol..

[8] Bornstein P. (2001). Thrombospondins as matricellular modulators of cell function. J. Clin. Investig..

[9] Carlson C.B., Lawler J., Mosher D.F. (2008). Structures of thrombospondins. Cell. Mol. Life Sci. CMLS.

[10] Narouz-Ott L., Maurer P., Nitsche D.P., Smyth N., Paulsson M. (2000). Thrombospondin-4 binds specifically to both collagenous and non-collagenous extracellular matrix proteins via its C-terminal domains. J. Biol. Chem..

[11] Adams J.C. (2004). Functions of the conserved thrombospondin carboxy-terminal cassette in cell–extracellular matrix interactions and signaling. Int. J. Biochem. Cell Biol..

[12] Arber S., Caroni P. (1995). Thrombospondin-4, an extracellular matrix protein expressed in the developing and adult nervous system promotes neurite outgrowth. J. Cell Biol..

[13] Stenina O.I., Desai S.Y., Krukovets I., Kight K., Janigro D., Topol E.J., Plow E.F. (2003). Thrombospondin-4 and its variants: Expression and differential effects on endothelial cells. Circulation.

[14] Forster S., Gretschel S., Jons T., Yashiro M., Kemmner W. (2011). THBS4, a novel stromal molecule of diffuse-type gastric adenocarcinomas, identified by transcriptome-wide expression profiling. Mod. Pathol. An Off. J. United States Can. Acad. Pathol. Inc..

[15] Liu J., Cheng G., Yang H., Deng X., Qin C., Hua L., Yin C. (2016). Reciprocal regulation of long noncoding RNAs THBS4003 and THBS4 control migration and invasion in prostate cancer cell lines. Mol. Med. Rep..

[16] McCart Reed A.E., Song S., Kutasovic J.R., Reid L.E., Valle J.M., Vargas A.C., Smart C.E., Simpson P.T. (2013). Thrombospondin-4 expression is activated during the stromal response to invasive breast cancer. Virchows Arch. An Int. J. Pathol..

[17] Lee J., Lee W.K., Seol M.-Y., Lee S.G., Kim D., Kim H., Park J., Jung S.G., Chung W.Y., Lee E.J. (2016). Coupling of LETM1 up-regulation with oxidative phosphorylation and platelet-derived growth factor receptor signaling via YAP1 transactivation. Oncotarget.

[18] Heldin C.H., Westermark B. (1999). Mechanism of action and in vivo role of platelet-derived growth factor. Physiol. Rev..

[19] Ostman A., Heldin C.H. (2007). PDGF receptors as targets in tumor treatment. Adv. Cancer Res..

[20] Hwang R.F., Yokoi K., Bucana C.D., Tsan R., Killion J.J., Evans D.B., Fidler I.J. (2003). Inhibition of platelet-derived growth factor receptor phosphorylation by STI571 (Gleevec) reduces growth and metastasis of human pancreatic carcinoma in an orthotopic nude mouse model. Clin. Cancer Res. An Off. J. Am. Assoc. Cancer Res..

[21] Maass T., Thieringer F.R., Mann A., Longerich T., Schirmacher P., Strand D., Hansen T., Galle P.R., Teufel A., Kanzler S. (2011). Liver specific overexpression of platelet-derived growth factor-B accelerates liver cancer development in chemically induced liver carcinogenesis. Int. J. Cancer.

[22] Seymour L., Bezwoda W.R. (1994). Positive immunostaining for platelet derived growth factor (PDGF) is an adverse prognostic factor in patients with advanced breast cancer. Breast Cancer Res. Treat..

[23] Lindmark G., Sundberg C., Glimelius B., Pahlman L., Rubin K., Gerdin B. (1993). Stromal expression of platelet-derived growth factor beta-receptor and platelet-derived growth factor B-chain in colorectal cancer. Lab. Investig. A J. Tech. Methods Pathol..

[24] Sundberg C., Ljungstrom M., Lindmark G., Gerdin B., Rubin K. (1993). Microvascular pericytes express platelet-derived growth factor-beta receptors in human healing wounds and colorectal adenocarcinoma. Am. J. Pathol..

[25] Wehler T.C., Frerichs K., Graf C., Drescher D., Schimanski K., Biesterfeld S., Berger M.R., Kanzler S., Junginger T., Galle P.R. (2008). PDGFRalpha/beta expression correlates with the metastatic behavior of human colorectal cancer: A possible rationale for a molecular targeting strategy. Oncol. Rep..

[26] Steller E.J., Ritsma L., Raats D.A., Hoogwater F.J., Emmink B.L., Govaert K.M., Laoukili J., Rinkes I.H., van Rheenen J., Kranenburg O. (2011). The death receptor CD95 activates the cofilin pathway to stimulate tumour cell invasion. EMBO Rep..

[27] Andrae J., Gallini R., Betsholtz C. (2008). Role of platelet-derived growth factors in physiology and medicine. Genes Dev..

[28] Fantauzzo K.A., Soriano P. (2016). PDGFRbeta regulates craniofacial development through homodimers and functional heterodimers with PDGFRalpha. Genes Dev..

[29] Chen J., Yuan W., Wu L., Tang Q., Xia Q., Ji J., Liu Z., Ma Z., Zhou Z., Cheng Y. (2017). PDGF-D promotes cell growth, aggressiveness, angiogenesis and EMT transformation of colorectal cancer by activation of Notch1/Twist1 pathway. Oncotarget.

[30] Jiang B., Chen J., Yuan W., Ji J., Liu Z., Wu L., Tang Q., Shu X. (2018). Platelet-derived growth factor-D promotes colorectal cancer cell migration, invasion and proliferation by regulating Notch1 and matrix metalloproteinase-9. Oncol. Lett..

[31] Muppala S., Frolova E., Xiao R., Krukovets I., Yoon S., Hoppe G., Vasanji A., Plow E., Stenina-Adognravi O. (2015). Proangiogenic properties of Thrombospondin-4. Arterioscler. Thromb. Vasc. Biol..

[32] Muppala S., Xiao R., Krukovets I., Verbovetsky D., Yendamuri R., Habib N., Raman P., Plow E., Stenina-Adognravi O. (2017). Thrombospondin-4 mediates TGF-beta-induced angiogenesis. Oncogene.

[33] Steller E.J., Raats D.A., Koster J., Rutten B., Govaert K.M., Emmink B.L., Snoeren N., van Hooff S.R., Holstege F.C., Maas C. (2013). PDGFRB promotes liver metastasis formation of mesenchymal-like colorectal tumor cells. Neoplasia.

[34] Anz D., Mueller W., Golic M., Kunz W.G., Rapp M., Koelzer V.H., Ellermeier J., Ellwart J.W., Schnurr M., Bourquin C. (2011). CD103 is a hallmark of tumor-infiltrating regulatory T cells. Int. J. Cancer.

[35] Lebrun J.J. (2012). The dual role of TGFbeta in human cancer: From tumor suppression to cancer metastasis. ISRN Mol. Biol..

[36] Tian J., Al-Odaini A.A., Wang Y., Korah J., Dai M., Xiao L., Ali S., Lebrun J.J. (2018). KiSS1 gene as a novel mediator of TGFbeta-mediated cell invasion in triple negative breast cancer. Cell. Signal..

[37] Bornfeldt K.E., Raines E.W., Graves L.M., Skinner M.P., Krebs E.G., Ross R. (1995). Platelet-derived growth factor. Distinct signal transduction pathways associated with migration versus proliferation. Ann. N. Y. Acad. Sci..

[38] Heldman A.W., Kandzari D.E., Tucker R.W., Crawford L.E., Fearon E.R., Koblan K.S., Goldschmidt-Clermont P.J. (1996). EJ-Ras inhibits phospholipase C gamma 1 but not actin polymerization induced by platelet-derived growth factor-BB via phosphatidylinositol 3-kinase. Circ. Res..

[39] Pinzani M. (2002). PDGF and signal transduction in hepatic stellate cells. Front. Biosci..

[40] Yao H., Duan M., Yang L., Buch S. (2012). Platelet-derived growth factor-BB restores human immunodeficiency virus Tat-cocaine-mediated impairment of neurogenesis: Role of TRPC1 channels. J. Neurosci..

[41] Pérez-Riesgo E., Gutiérrez L.G., Ubierna D., Acedo A., Moyer M.P., Núñez L., Villalobos C. (2017). Transcriptomic analysis of calcium remodeling in colorectal cancer. Int. J. Mol. Sci..

[42] Prevarskaya N., Ouadid-Ahidouch H., Skryma R., Shuba Y. (2014). Remodelling of Ca2+ transport in cancer: How it contributes to cancer hallmarks?. Philos. Trans. Royal Soc. B Biol. Sci..

[43] Stewart T.A., Yapa K.T., Monteith G.R. (2015). Altered calcium signaling in cancer cells. Biochim. Biophys. Acta (BBA)-Biomembr..

[44] Bergsten E., Uutela M., Li X., Pietras K., Ostman A., Heldin C.H., Alitalo K., Eriksson U. (2001). PDGF-D is a specific, protease-activated ligand for the PDGF beta-receptor. Nat. Cell Biol..

[45] Greco S.A., Chia J., Inglis K.J., Cozzi S.J., Ramsnes I., Buttenshaw R.L., Spring K.J., Boyle G.M., Worthley D.L., Leggett B.A. (2010). Thrombospondin-4 is a putative tumour-suppressor gene in colorectal cancer that exhibits age-related methylation. BMC Cancer.

[46] Thomson S., Petti F., Sujka-Kwok I., Epstein D., Haley J.D. (2008). Kinase switching in mesenchymal-like non-small cell lung cancer lines contributes to EGFR inhibitor resistance through pathway redundancy. Clin. Exp. Metastasis.

[47] Jechlinger M., Sommer A., Moriggl R., Seither P., Kraut N., Capodiecci P., Donovan M., Cordon-Cardo C., Beug H., Grunert S. (2006). Autocrine PDGFR signaling promotes mammary cancer metastasis. J. Clin. Investig..

[48] Katz L.H., Li Y., Chen J.S., Munoz N.M., Majumdar A., Chen J., Mishra L. (2013). Targeting TGF-beta signaling in cancer. Expert Opin. Ther. Targets.

[49] Wang Z., Kong D., Banerjee S., Li Y., Adsay N.V., Abbruzzese J., Sarkar F.H. (2007). Down-regulation of platelet-derived growth factor-D inhibits cell growth and angiogenesis through inactivation of Notch-1 and nuclear factor-kappaB signaling. Cancer Res..

[50] Cui C., Merritt R., Fu L., Pan Z. (2017). Targeting calcium signaling in cancer therapy. Acta Pharm. Sin. B.

[51] Csordas G., Varnai P., Golenar T., Roy S., Purkins G., Schneider T.G., Balla T., Hajnoczky G. (2010). Imaging interorganelle contacts and local calcium dynamics at the ER-mitochondrial interface. Mol. Cell.

[52] Varnai P., Balla A., Hunyady L., Balla T. (2005). Targeted expression of the inositol 1,4,5-triphosphate receptor (IP3R) ligand-binding domain releases Ca2+ via endogenous IP3R channels. Proc. Natl. Acad. Sci. USA.

[53] Kondratskyi A., Yassine M., Kondratska K., Skryma R., Slomianny C., Prevarskaya N. (2013). Calcium-permeable ion channels in control of autophagy and cancer. Front. Physiol..

[54] Vandewalle B., Hornez L., Wattez N., Revillion F., Lefebvre J. (1995). Vitamin-D3 derivatives and breast-tumor cell growth: Effect on intracellular calcium and apoptosis. Int. J. Cancer.

[55] Liu L.H., Boivin G.P., Prasad V., Periasamy M., Shull G.E. (2001). Squamous cell tumors in mice heterozygous for a null allele of Atp2a2, encoding the sarco(endo)plasmic reticulum Ca2+-ATPase isoform 2 Ca2+ pump. J. Biol. Chem..

[56] Roderick H.L., Cook S.J. (2008). Ca2+ signalling checkpoints in cancer: Remodelling Ca2+ for cancer cell proliferation and survival. Nat. Rev. Cancer.

[57] Karacosta L.G., Foster B.A., Azabdaftari G., Feliciano D.M., Edelman A.M. (2012). A regulatory feedback loop between Ca2+/calmodulin-dependent protein kinase kinase 2 (CaMKK2) and the androgen receptor in prostate cancer progression. J. Biol. Chem..

[58] Monteith G.R., Davis F.M., Roberts-Thomson S.J. (2012). Calcium channels and pumps in cancer: Changes and consequences. J. Biol. Chem..

[59] Lawler J., Connolly J.E., Ferro P., Derick L.H. (1986). Thrombin and chymotrypsin interactions with thrombospondin. Ann. New York Acad. Sci..

[60] McLaughlin J.N., Mazzoni M.R., Cleator J.H., Earls L., Perdigoto A.L., Brooks J.D., Muldowney J.A., Vaughan D.E., Hamm H.E. (2005). Thrombin modulates the expression of a set of genes including thrombospondin-1 in human microvascular endothelial cells. J. Biol. Chem..

[61] Baenziger N.L., Brodie G.N., Majerus P.W. (1971). A thrombin-sensitive protein of human platelet membranes. Proc. Natl. Acad. of Sci. USA.

