# Supplementary material for: Potential Role of PDGFRβ-Associated THBS4 in Colorectal Cancer Development
Source: Cancers (Basel). 2020 Sep 6;12(9):2533. doi: 10.3390/cancers12092533 (PMC7564555; doi:10.3390/cancers12092533)
Supplement: Supplementary file 1 [file cancers-12-02533-s001.pdf]

Supplementary Materials

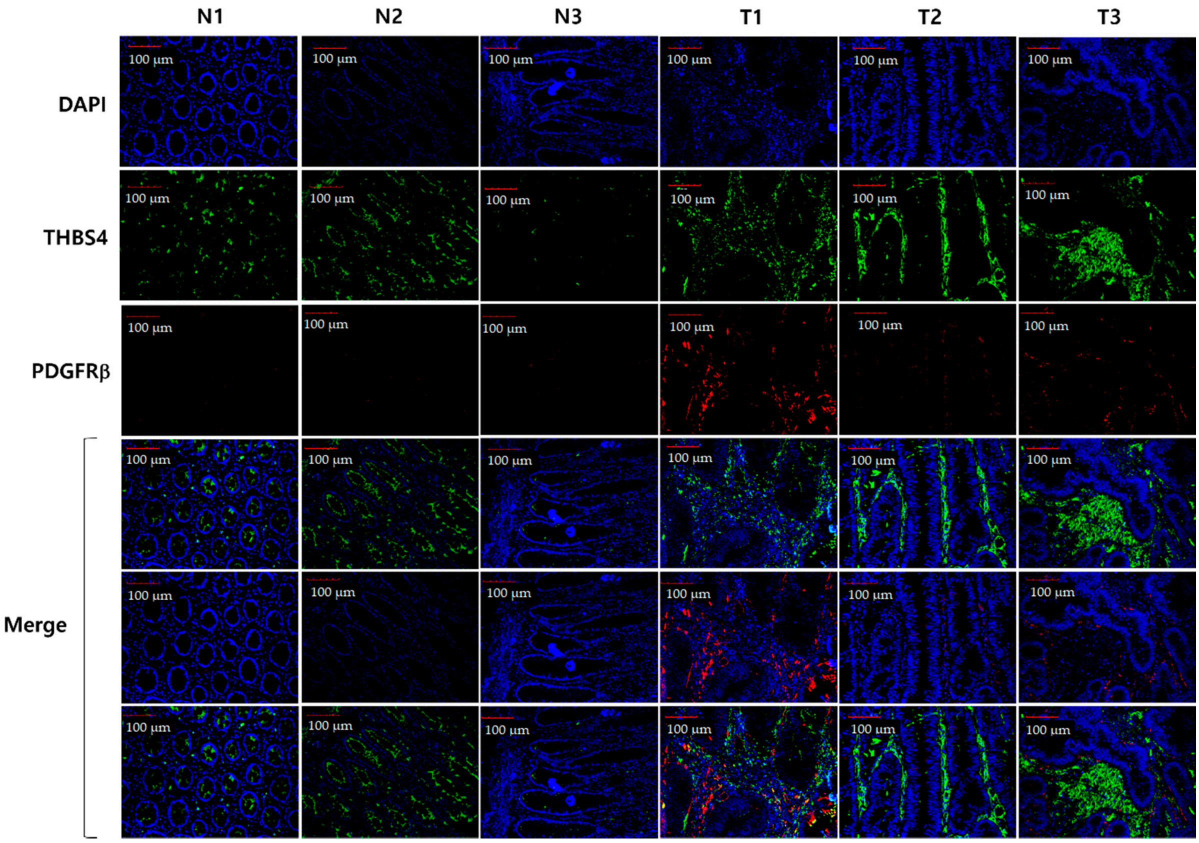

Figure S1. Co-immunofluorescence and immunoblots for THBS4 and PDGFRβ in normal and tumor tissues of colon cancer patients. Immunofluorescence with an anti-THBS4 antibody (green) and anti-PDGFRβ antibody (red) on normal and tumor tissues of colon cancer patients. Scale bars = 100 μm.

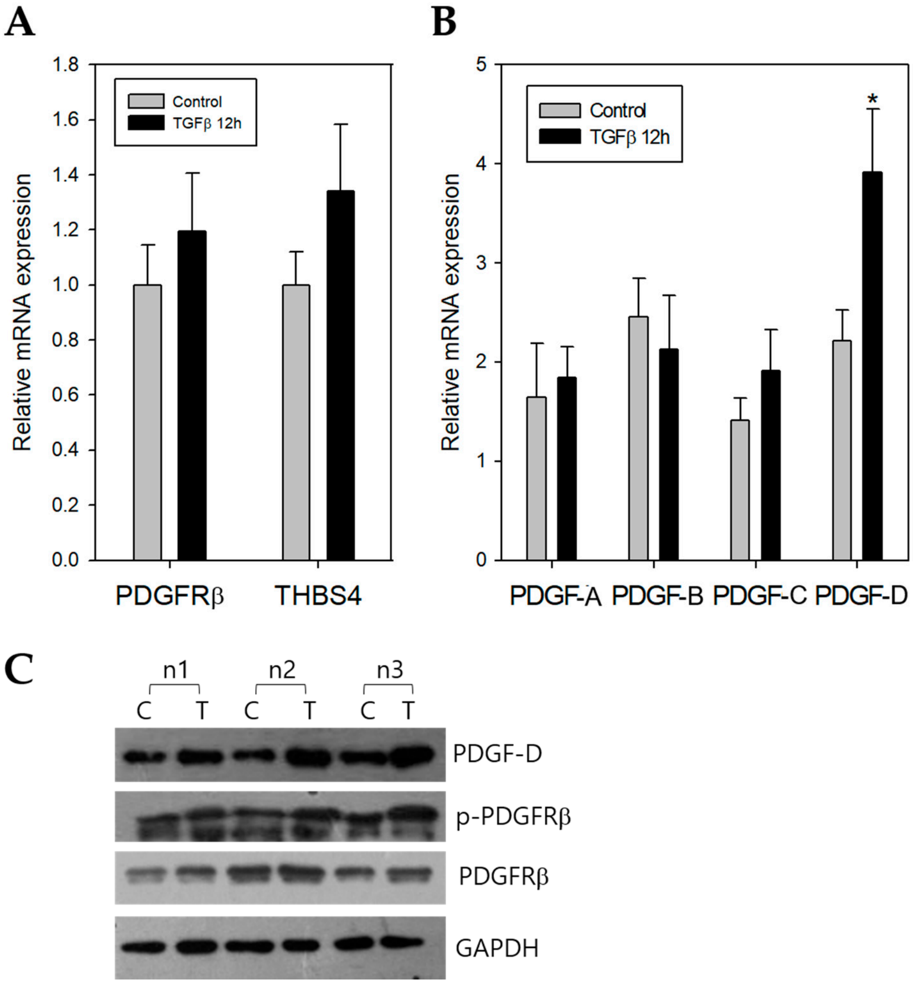

Figure S2. Effect of TGFβ on mRNA levels of THBS4, PDGFRβ, and PDGFRβ ligands. Relative mRNA expression levels as determined with real-time PCR for (**A**) THBS4, PDGFRβ, (**B**) PDGF-A, PDGF-B, PDGF-C, and PDGF-D of HCT116 cells cultured in the presence (TGFβ 12 h) or absence (Control) of TGFβ (10 μM) for 12 h. (**C**) Western blot with anti-PDGF-D, p-PDGFRβ, and PDGFRβ antibodies in DLD-1 cells cultured in the presence (TGFβ 10 μM) or absence (control) of TGFβ for 12 h. Three independent experiments were performed in duplicate. * $p < 0.05$, compared with control, *t*-test.

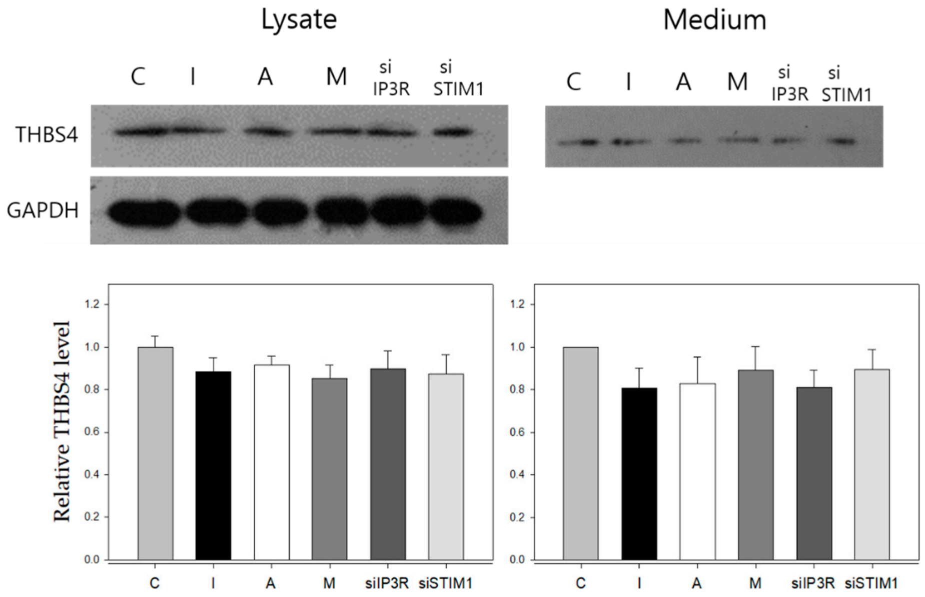

Figure S3. Protein levels of THBS4 without PDGF-D stimulation. Western blot with anti-THBS4 antibody in whole cell lysate (left panel) or cultured medium (right panel) of DLD-1 cells cultured in the presence of imatinib 5 μM for 24 h, 2-APB 100 μM for 24 h, or ML-9 100 μM for 24 h, respectively. Three independent experiments were performed in duplicate.

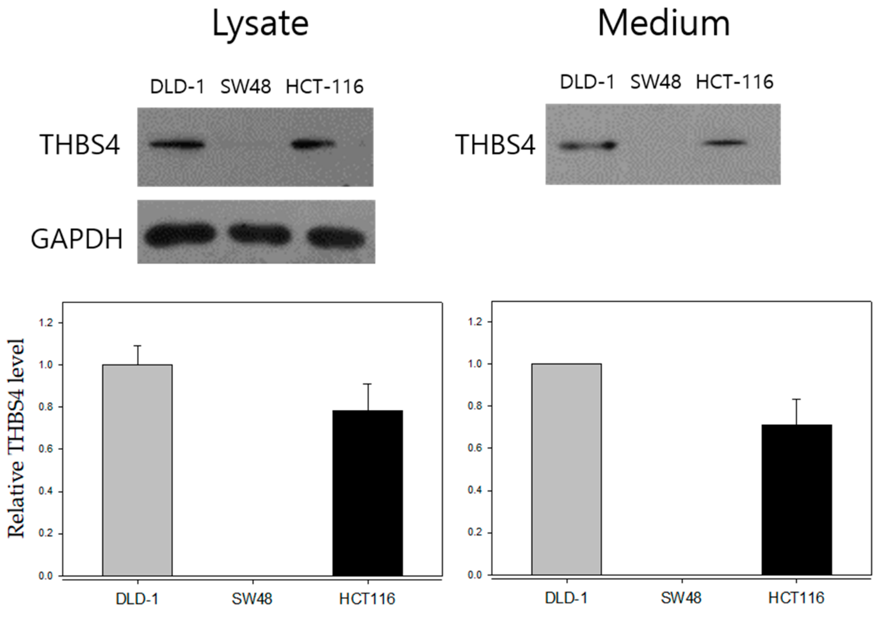

Figure S4. Basal THBS4 levels in DLD-1, SW40, and HCT-116 cells. Western blot with anti-THBS4 antibody in whole cell lysate (left panel) or cultured medium (right panel) of DLD-1, SW48, and HCT-116 cells. Three independent experiments were performed in duplicate.

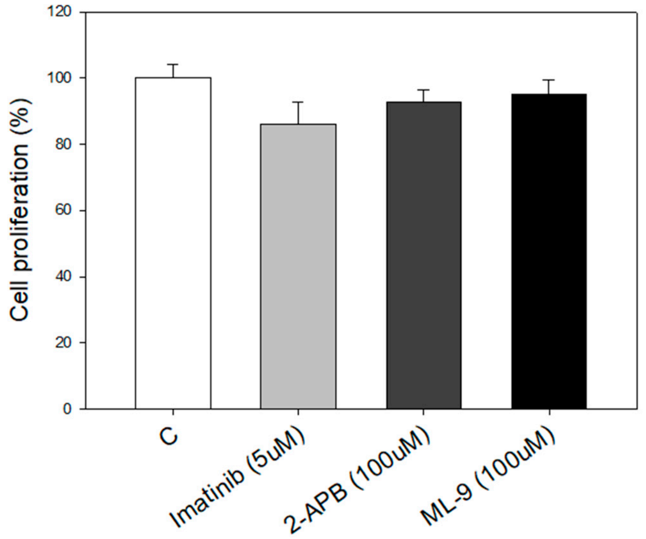

Figure S5. Cell proliferation of DLD-1 cells after treatment with imatinib, 2-APB, and ML-9. DLD-1 cells were cultured with imatinib 5 μM, 2-APB 100 μM, or ML-9 100 μM for 24 h and subjected to an MTT assay. Three independent experiments were performed in duplicate.

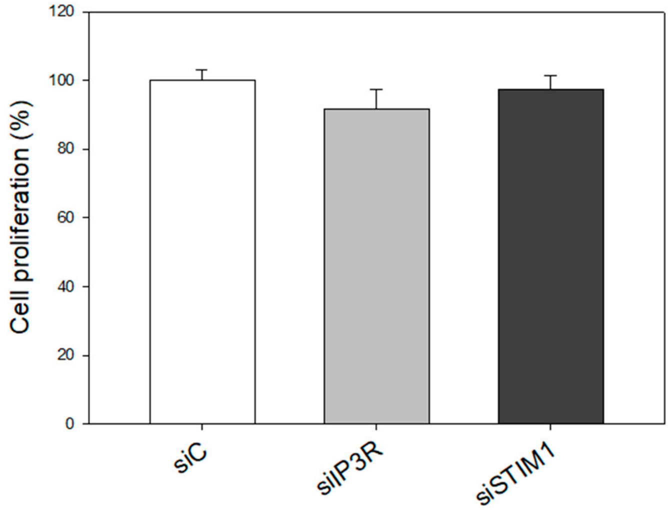

Figure S6. Cell proliferation of DLD-1 cells after siIP3R and siSTIM1 transfection. DLD-1 cells were transfected with siIP3R and siSTIM1 and subjected to an MTT assay. Three independent experiments were performed in duplicate.

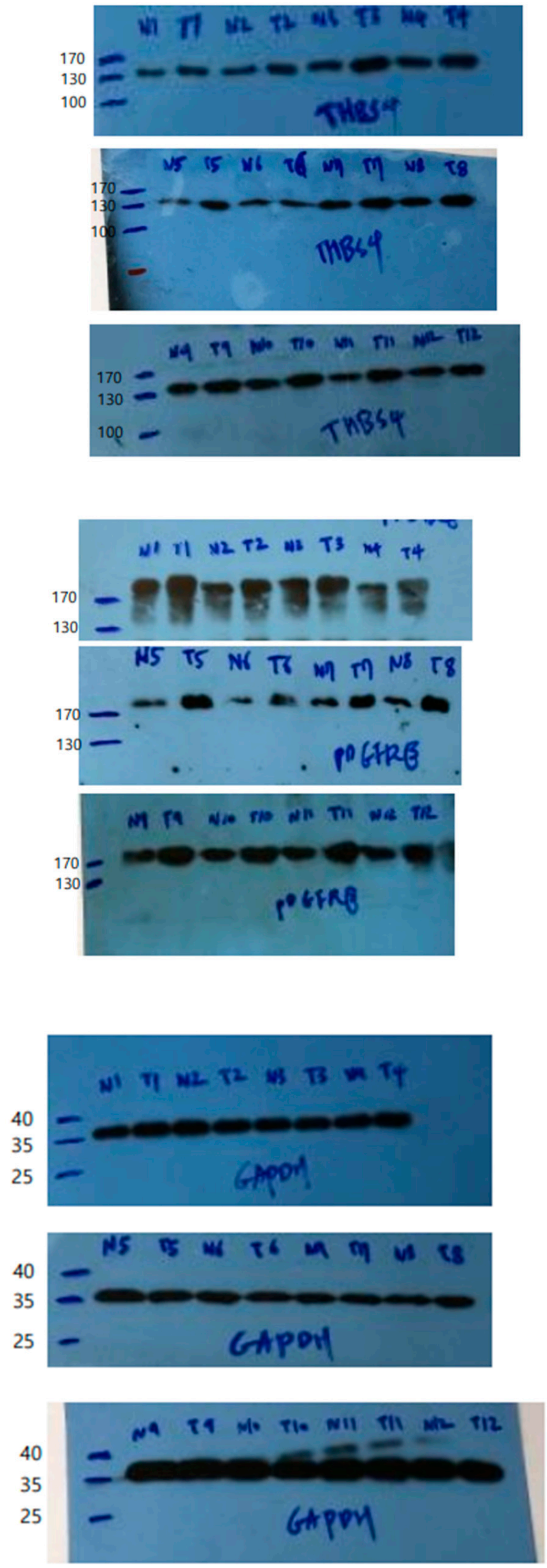

Figure S7. Original Western Blots of figure 1.

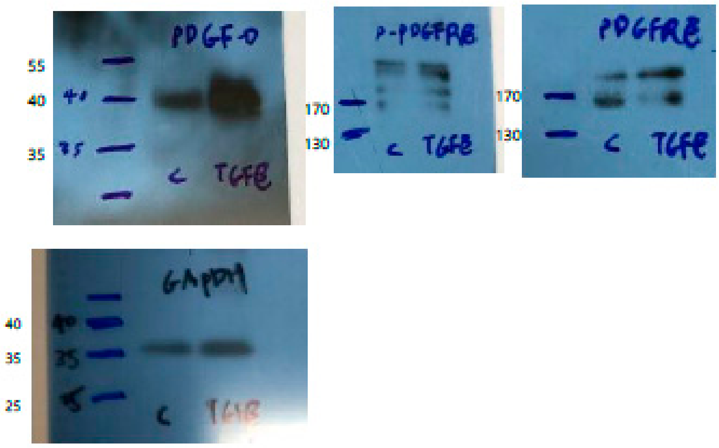

Figure S8. Original Western Blots of figure 2.

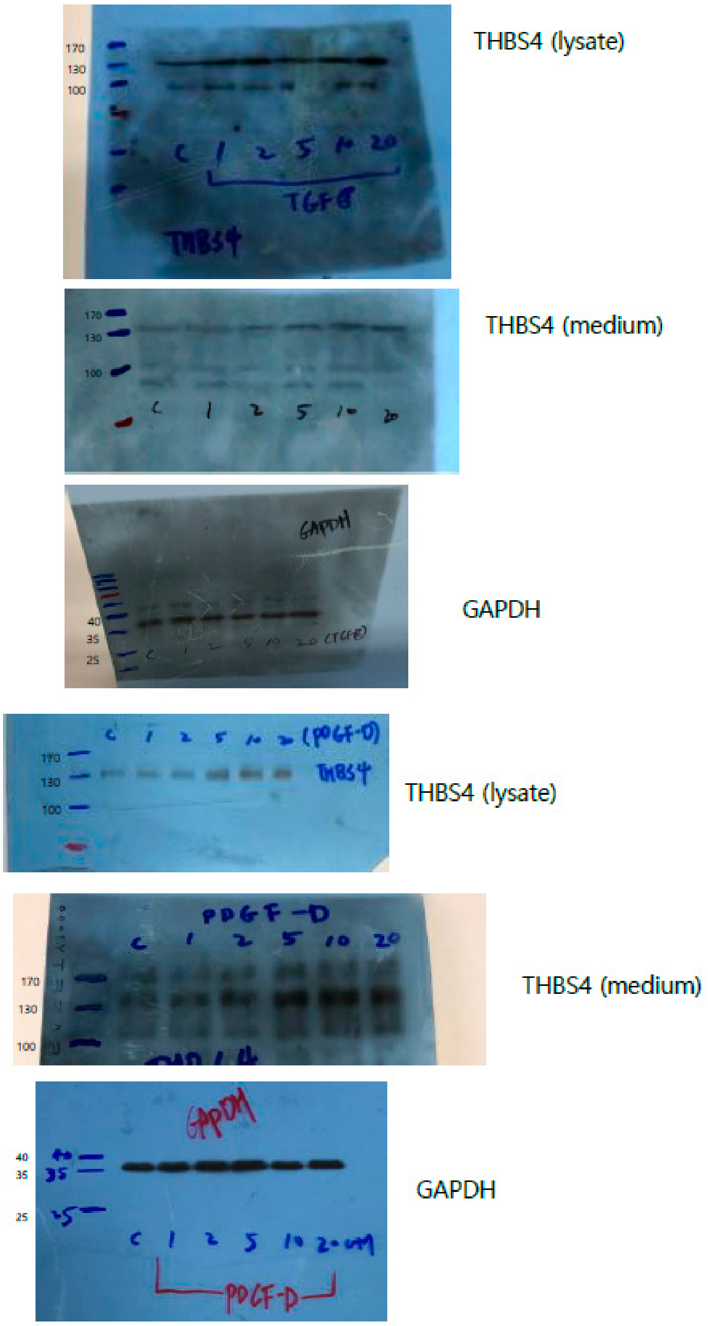

Figure S9. Original Western Blots of figure 3.

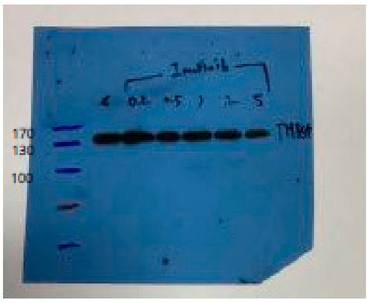

THBS4 (lysate)

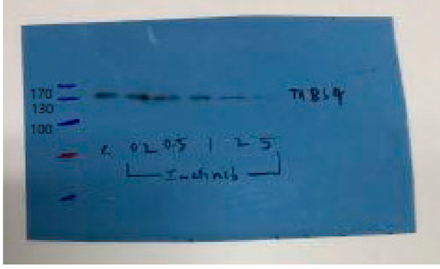

THBS4 (medium)

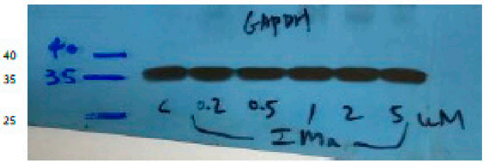

GAPDH

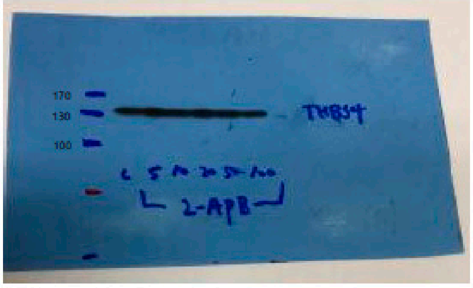

THBS4 (lysate)

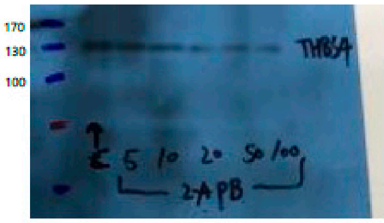

THBS4 (medium)

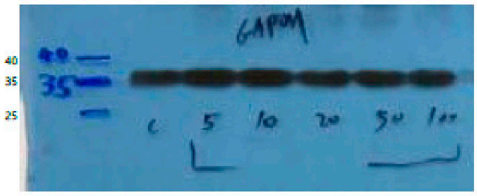

GAPDH

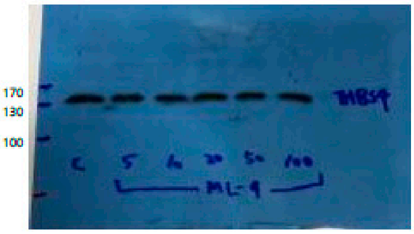

THBS4 (lysate)

Figure S10. *Cont.*

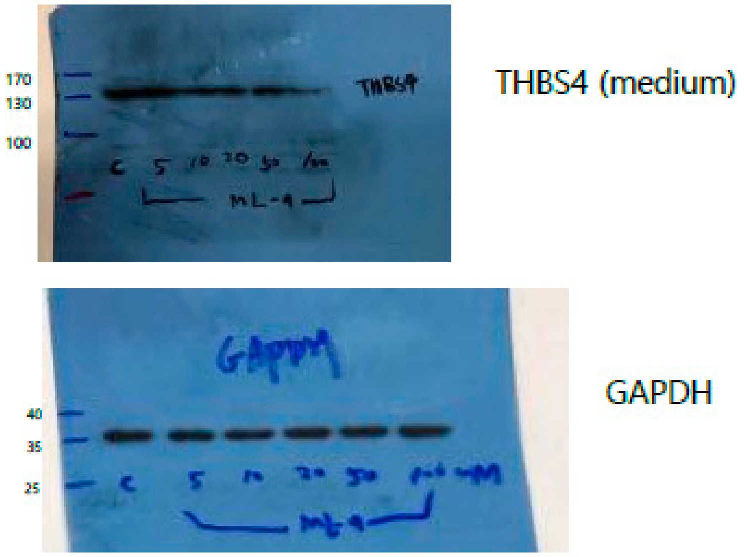

Figure S10. Original Western Blots of figure 4A.

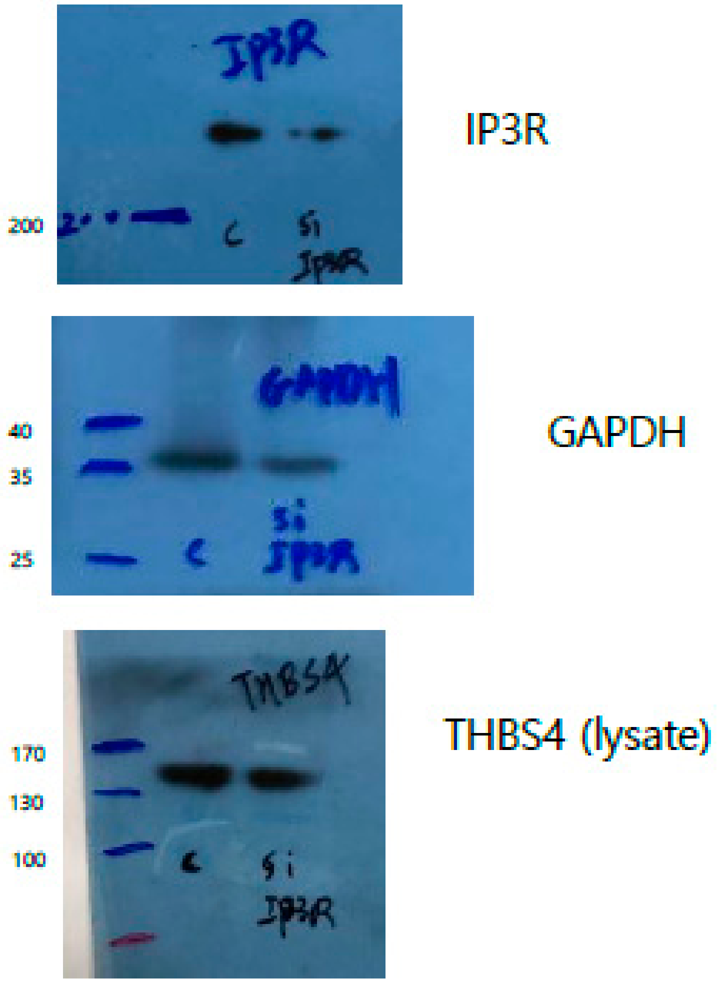

Figure S11. *Cont.*

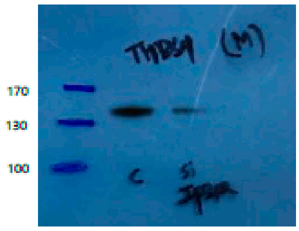

THBS4 (medium)

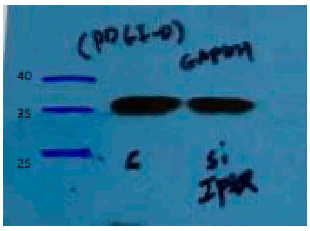

GAPDH

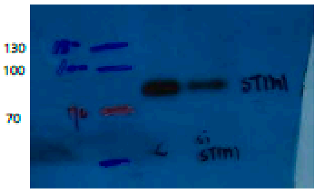

STIM1

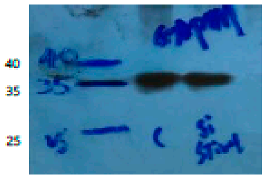

GAPDH

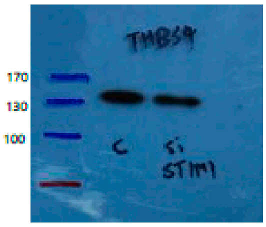

THBS4 (lysate)

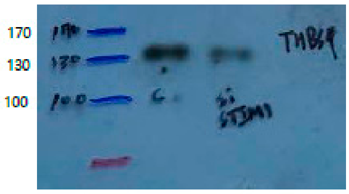

THBS4 (medium)

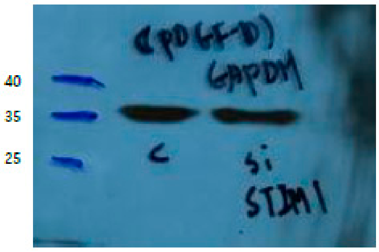

GAPDH

Figure S11. Original Western Blots of figure 4B.

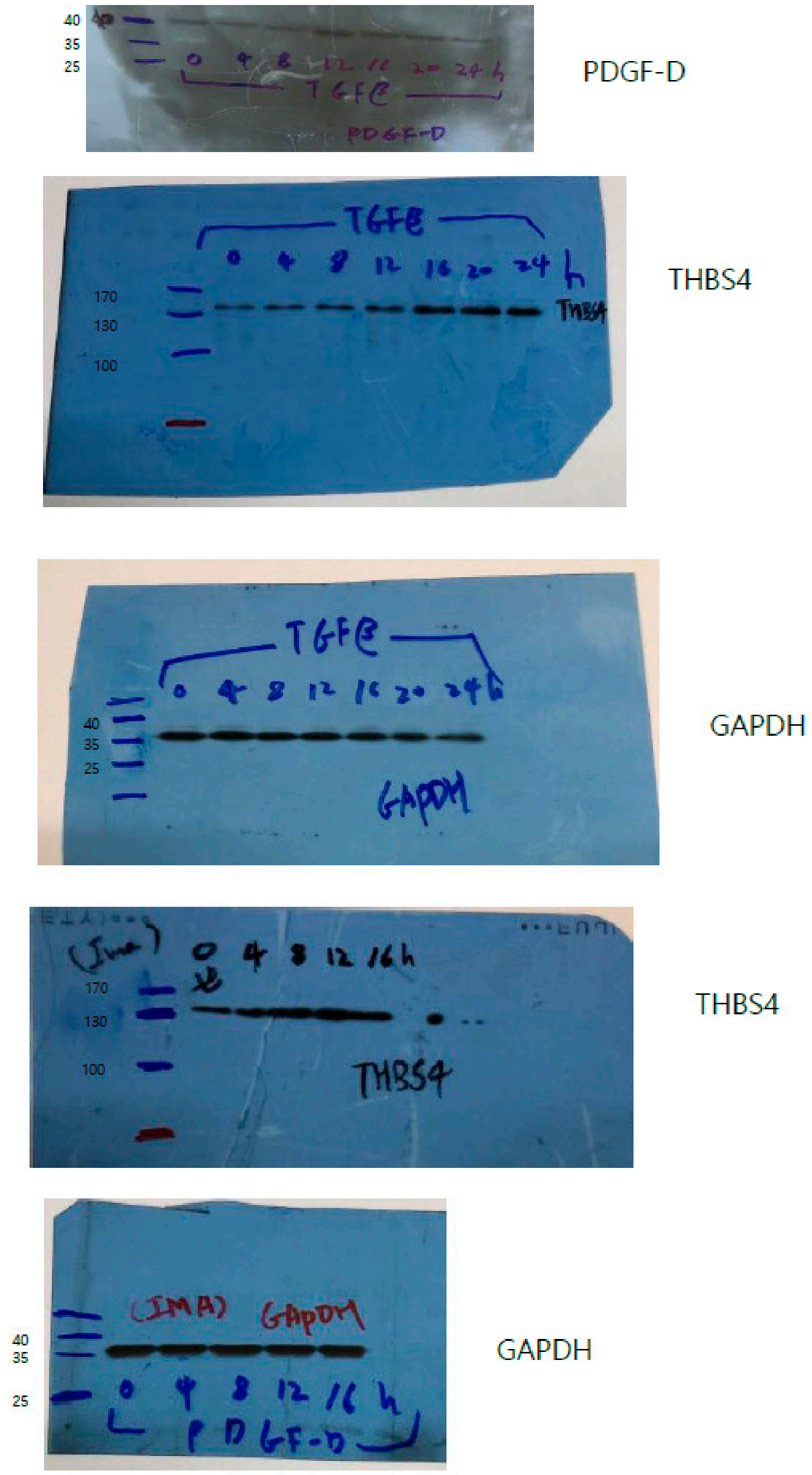

Figure S12. Original Western Blots of figure 5A.

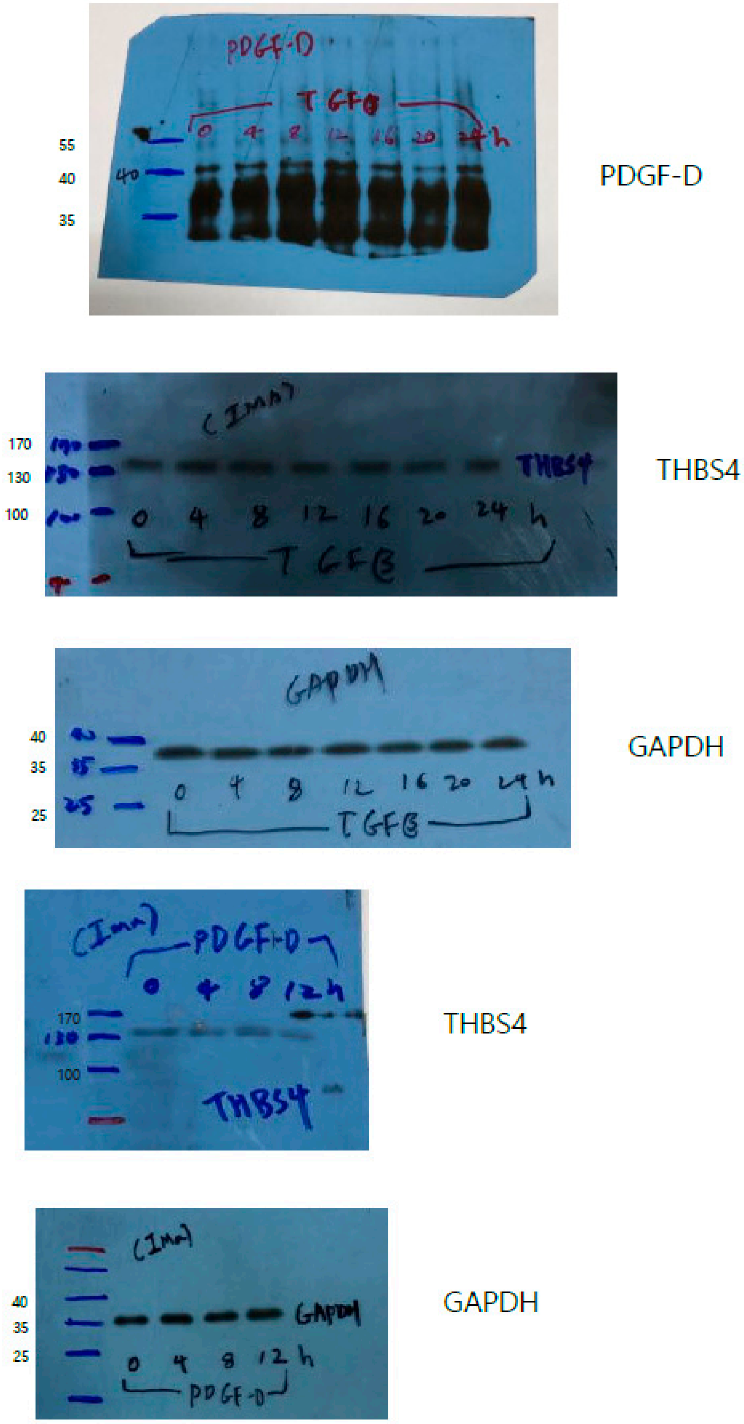

Figure S13. Original Western Blots of figure 5B.

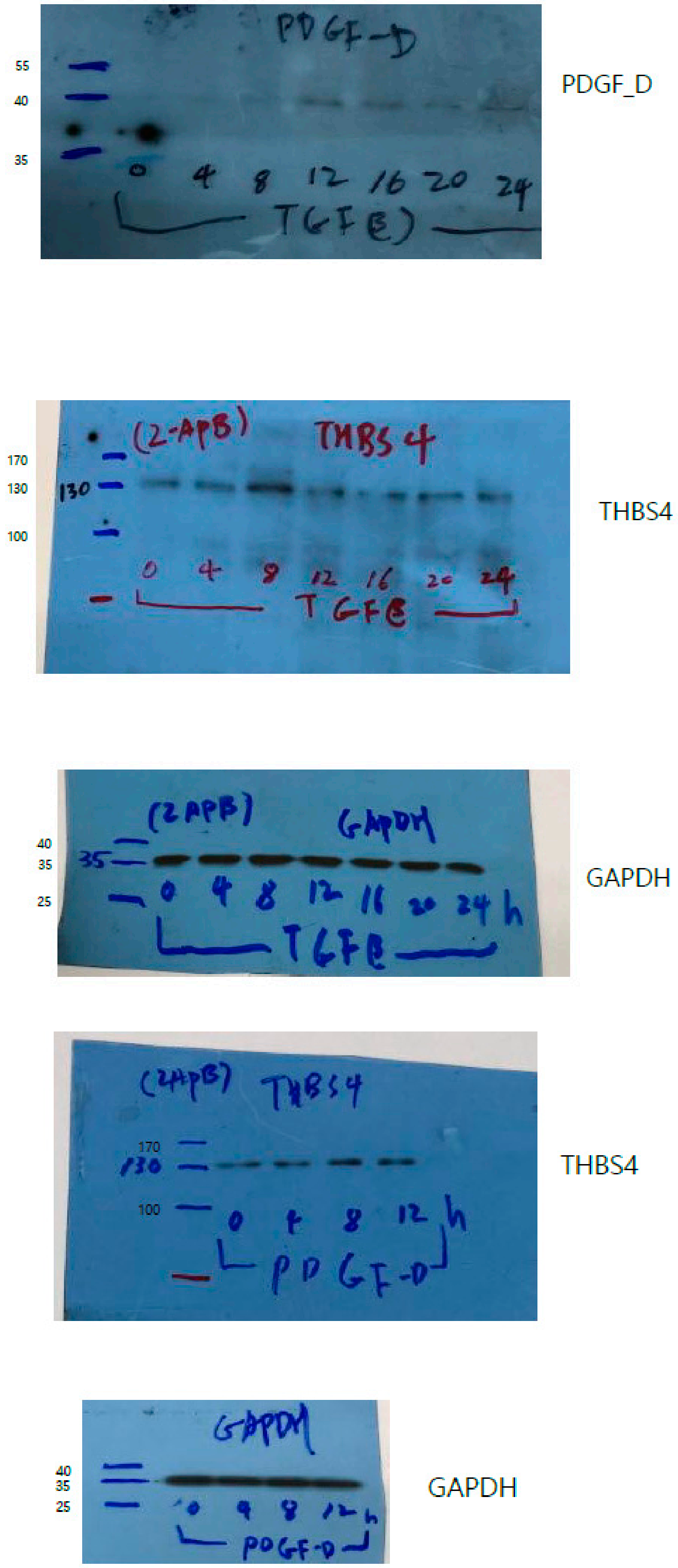

Figure S14. Original Western Blots of figure 5C.
